# Facile fabrication of superhydrophobic surfaces with hierarchical structures

**DOI:** 10.1038/s41598-018-22501-8

**Published:** 2018-03-06

**Authors:** Eunyoung Lee, Kun-Hong Lee

**Affiliations:** Department of Chemical Engineering, Pohang University of Science and Technology, 77 Cheongam-Ro, Nam-Gu, Pohang, Gyeongbuk South Korea

## Abstract

Hierarchical structures were fabricated on the surfaces of SUS304 plates using a one-step process of direct microwave irradiation under a carbon dioxide atmosphere. The surface nanostructures were composed of chrome-doped hematite single crystals. Superhydrophobic surfaces with a water contact angle up to 169° were obtained by chemical modification of the hierarchical structures. The samples maintained superhydrophobicity under NaCl solution up to 2 weeks.

## Introduction

Hierarchical structures are a common feature of hydrophobic surfaces found in nature. For example, the lotus leaf, Salvinia molesta leaf, and legs of *amenbô* show the presence of hierarchical structures consisting of nanoscale wax protrusions on microscale roughness. Superhydrophobic surface requires surface roughness to decrease the contact area with water to the surface structure^1,2^. Hierarchical surface is known to be more beneficial than surface with mono-scale roughness for superhydrophobicity^3,4^. Different techniques for the fabrication of artificial superhydrophobic surfaces based on hierarchical structures have been developed in recent years^3–9^. Fabrication of this rough structure has been studied with various substrate, such as polymers^10–18^, silicon^19–24^, and metals^5,25–31^. To make an efficient superhydrophobic surface, not only the surface roughness should be increased, but also the surface energy should be lowered^32,33^. Polymers such as Teflon^34^ have low surface energy, so fabrication of surface having nano or micro-scale (or both) roughness with polymers can result in the superhydrophobic surface. Low surface energy is usually obtained with surface coating with chemicals having long alkyl or fluoro-alkyl chain such as self-assembled monolayer (SAM) coating^18^ or deposition of Teflon-like polymer film^17^.

There have been many studies to grow metal oxide nanostructure directly from bulk metal by surface oxidization, such as thermal heating^35–39^, resistive heating^40^ and plasma synthesis^41–44^. Since highly reactive radicals and reactive species are created inside the plasma, plasma oxidation has the advantage in reaction time and temperature compared to traditional thermal heating method. One-dimensional nanorod structure and two-dimensional nanobelts are most frequently seen in these methods with uniform nano-scale arrays. On the other hand, in this research, complex oxide nanostructures with dual-scale roughness were fabricated by one-step direct microwave irradiation to SUS304 surface. This method does not require e multiple steps to make micro-scale and nano-scale structures separately; just only direct microwave irradiation leads to the hierarchical structure to SUS304 surface. Moreover, this method needs very short reaction time due to rapid growth caused by plasma, and is expected to be easily scaled up.

**Figure 1:**
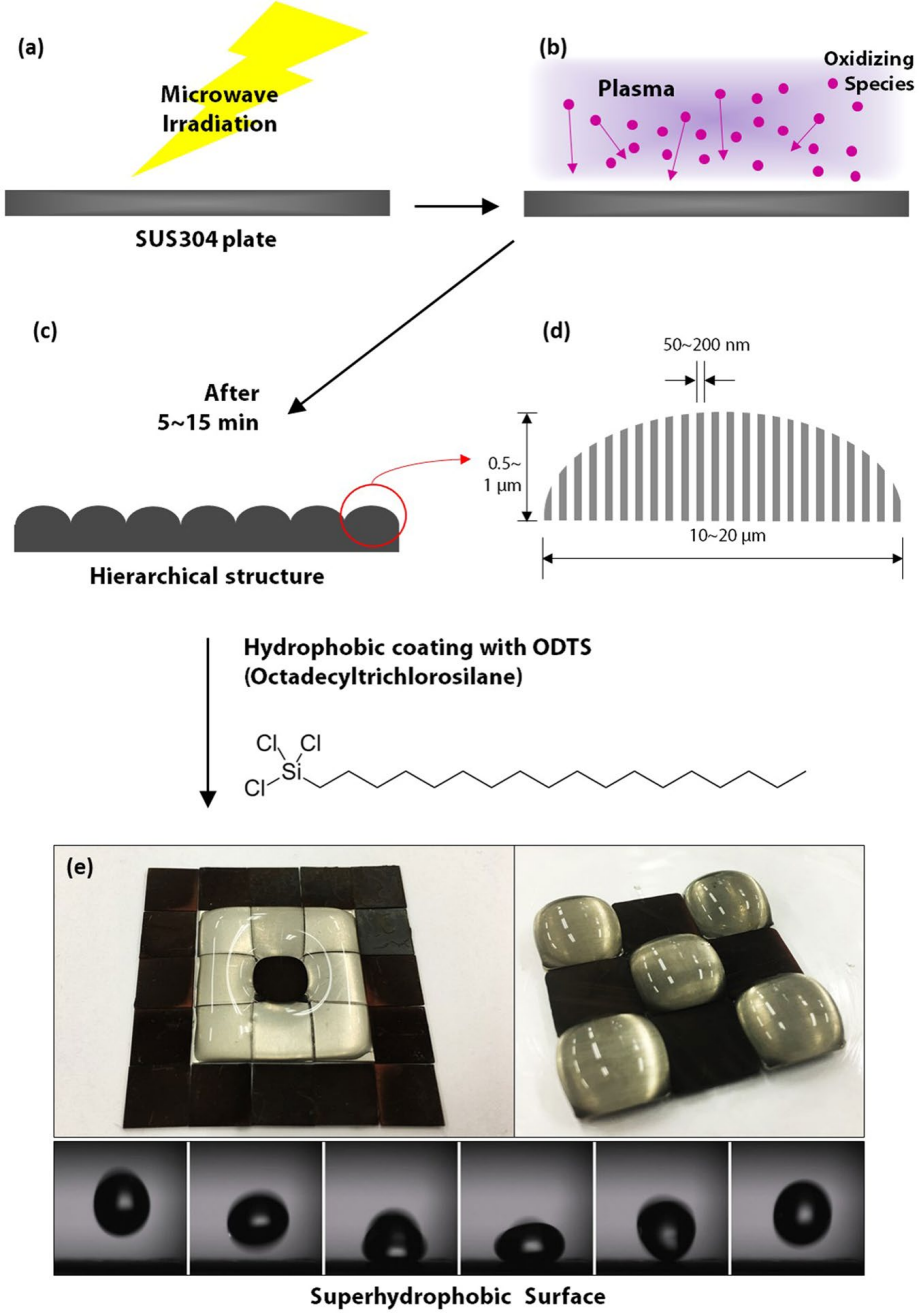
Schematic of the procedure for preparing the superhydrophobic surface with hierarchical structures. (**a**) Microwave irradiation of SUS304 plates under a low vacuum environment. (**b**) Generation of plasma and formation of oxidizing species inside the plasma, which bombard the surface. (**c**) Formation of hierarchical structures on the SUS304 plate after 5~15 minutes of irradiation. (**d**) Schematic of a single protrusion in the fabricated hierarchical structure. (**e**) Superhydrophobic surface obtained after ODTS coating. The black regions are superhydrophobic surfaces, and the gray regions represent unirradiated SUS304 plates (hydrophilic).

The surface with the fabricated hierarchical structures was transformed to a superhydrophobic surface with a simple coating of silane. The schematic of the procedure for the preparation of the superhydrophobic surface is shown in Fig. 1. Microwave irradiation of a stainless steel 304 (SUS304) plate under a low-vacuum CO_2_ atmosphere generates plasma with oxidizing species (Fig. 1b) that bombard the surface to create hierarchical structures consisting of microhills (10~20 μm base diameter) and nanonrods (0.5~1 μm in height and 50~200 nm in diameter) (Fig. 1c,d). Superhydrophobic surfaces, with water contact angles up to 169°, were prepared by coating the hierarchical structures with a hydrophobic coating (Fig. 1e; multiple samples were used).

## Results and Discussion

**Figure 2:**
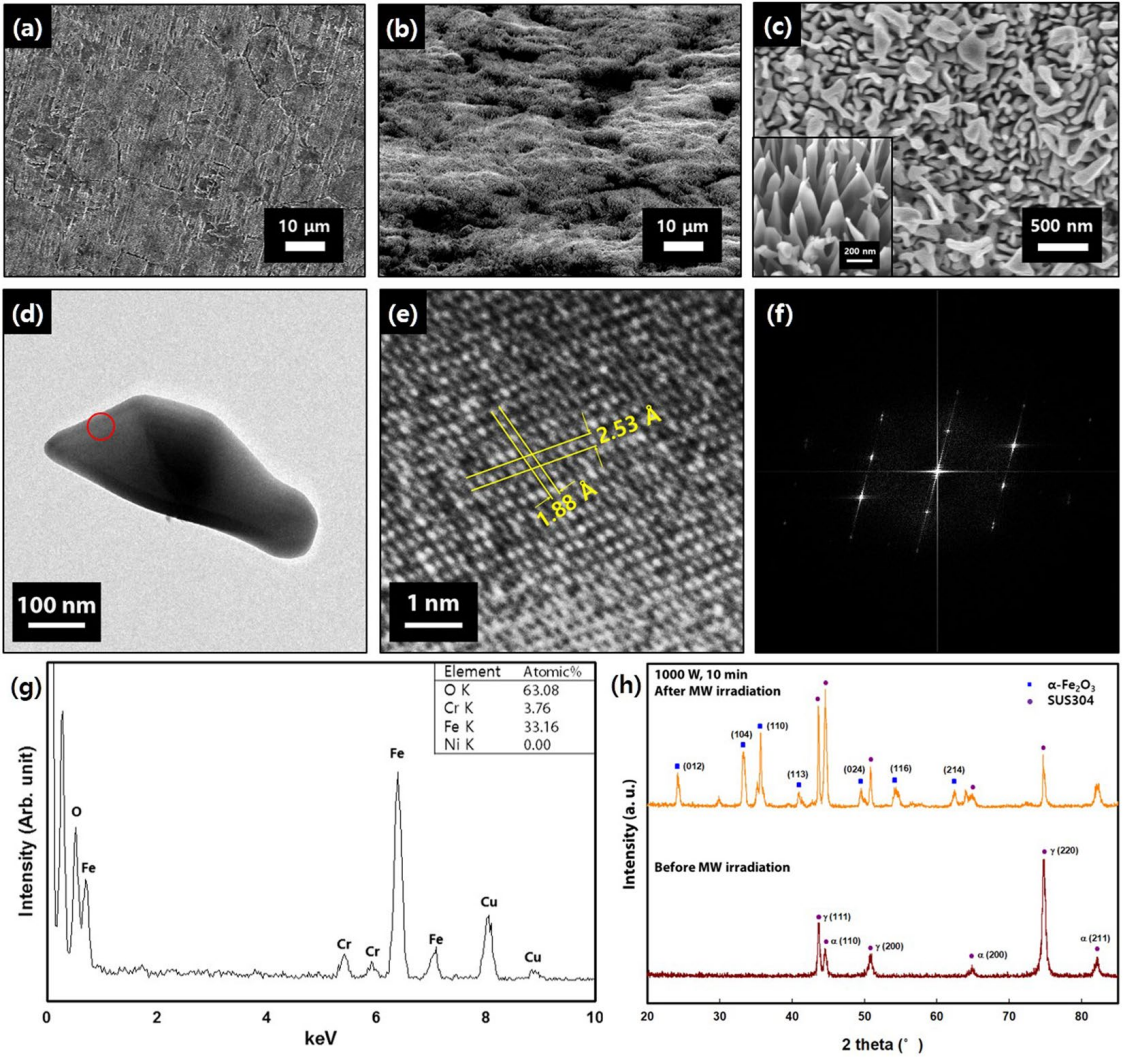
(**a**) SEM image of the surface of the pristine SUS304 plate before microwave irradiation. (**b**) SEM image showing the nanoscale protrusions on the surface of the SUS304 plate after microwave irradiation at 1500 W for 10 min. (**c**) Higher magnification SEM image of the surface of the same sample showing the nanostructures. The inset image shows the structure with higher magnification. (The inset image was taken from different sample with same condition, so the image could be seen a little different). (**d**) TEM image of a typical nanostructure. (**e**) High-resolution TEM image of the region marked by the red circle in (**d**). (**f**) FFT image obtained from (**e**). (**g**) EDS data of the region marked by the red circle in (**d**) (Here, the atomic concentrations shown are the average values of twenty different structures). (**h**) XRD data of the SUS304 plate before and after the microwave irradiation.

When the metal plate is subjected to microwave irradiation at a low pressure, the microwave interacts with the metal surface to cause a spark, and this spark generates plasma inside the reactor. In this study, CO_2_ was used as the working gas for the creation of the plasma, and the hierarchical structures were fabricated on the surfaces of SUS304 plates. Figure 2a shows the surface of the pristine SUS304 plate, showing no macroscopic roughness. Hierarchical structures consisting of both 10~100 μm-scale protrusions (Fig. 2b) and much smaller nanostructures (Fig. 2c) were formed after the microwave irradiation. The nanostructures were 200~500 nm in height and 50~200 nm in width. The individual nanostructures were observed by TEM, and the TEM image of a typical nanostructure is shown in Fig. 2d. This nanostructure is single-crystalline as evidenced by the high-resolution TEM image (Fig. 2e) and the fast Fourier transform (FFT) image (Fig. 2f). The EDS analysis (Fig. 2g) revealed that the composition of the nanostructure is Fe:Cr:O = 33:4:63. It is interesting to note that Ni was not detected after the microwave irradiation even though SUS304 is an alloy of Fe, Cr, and Ni. A plausible explanation is that the heat of formation of NiO (−58.1 kcal/mol) is higher than that of Cr_2_O_3_ (−252.9 kcal/mol) or Fe_2_O_3_ (−197.0 kcal/mol)^45^. Therefore, it is believed that Ni is harder to be oxidized and does not appear on the surface structure. However, further studies are required to elucidate this result. Figure 2h shows the XRD data of the SUS304 plate before and after the microwave irradiation. The XRD pattern of the pristine SUS304 plate showed peaks corresponding to the γ-phase and the α-phase. After the microwave irradiation, several peaks corresponding to α-Fe_2_O_3_ were observed. This suggests that the fabricated nanostructures are composed of mainly α-Fe_2_O_3_, containing small amounts of Cr inside the crystal. It is considered that some of the Fe sites were replaced by Cr atoms; since Fe^3+^ (0.785 Å) and Cr^3+^ (0.755 Å) ions are similar in size, the crystal units will remain almost the same, and the XRD peaks will be observed at approximately the same angles. Interestingly, peaks of other iron oxides, such as FeO and Fe_3_O_4_, were not observed in the XRD data. It has been reported that a 3-layer structure was synthesized by thermal oxidation of an iron sheet, due to oxygen diffusion^37,40,46^. However, in the present study, it is likely that only the Fe_2_O_3_ layer, which consists of the highest concentration of oxygen among the different iron oxides, was formed because of the high mobility of the O radicals inside the microwave plasma.

It is to be mentioned that carbon was not detected on the surfaces of the samples after the microwave irradiation. Dissociation and ionization processes are known to occur simultaneously inside the CO_2_ plasma, and dissociation is the predominant process. When irradiated by a microwave power of 700 W at 0.5 Torr, less than 10% of carbon dioxide is decomposed to form O radicals, which react with the surface to form oxide structures. CO_2_ → CO + O is the dominant reaction, and the CO radical is hardly decomposed^47^. As carbon radicals were not formed during the reaction, carbon was not detected on the surface.

**Figure 3:**
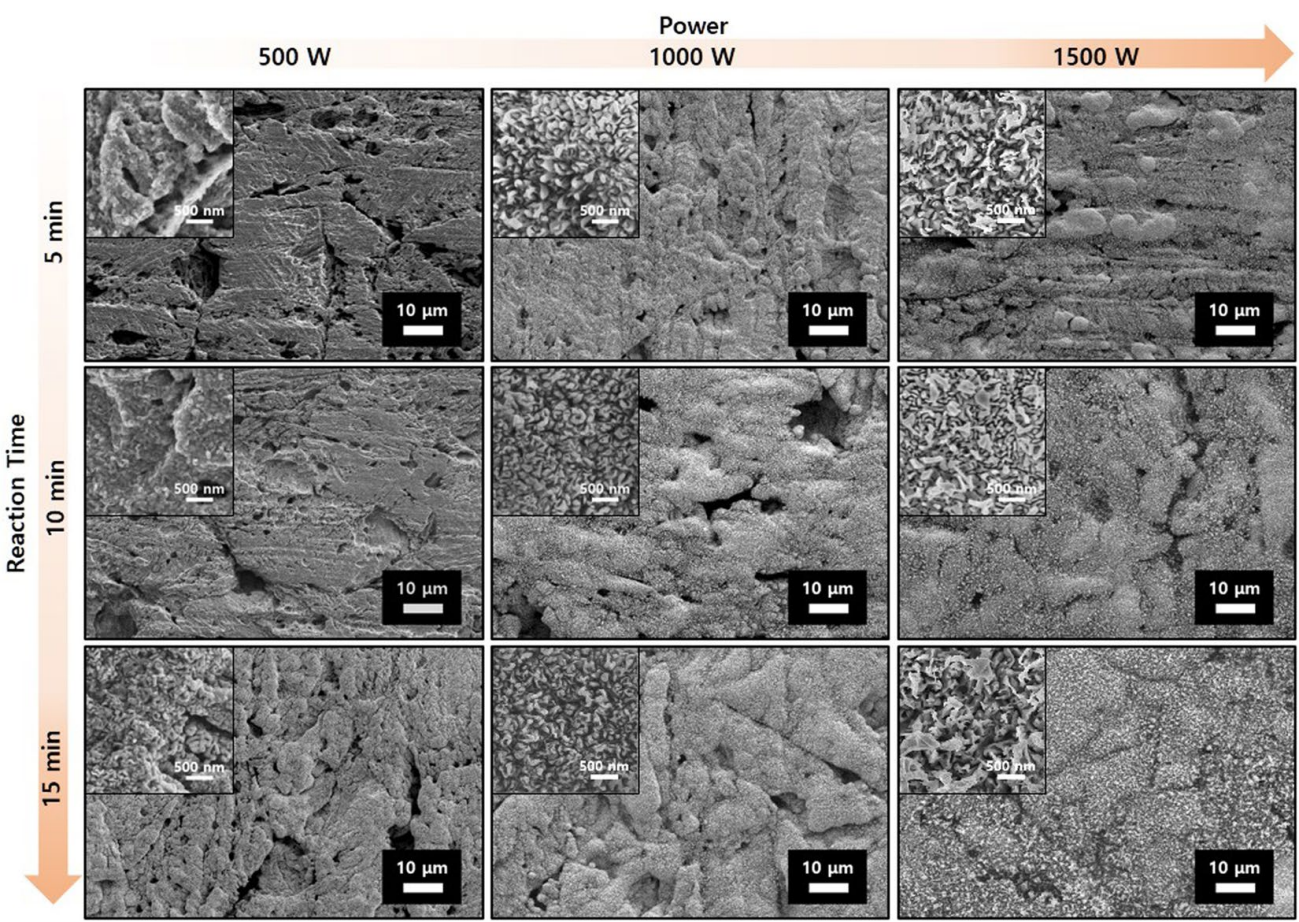
Variations in the surface morphology of the SUS 304 plate irradiated at different values of microwave power and irradiation time. Larger inset images with larger nanostructure views can be found in Fig. S5.

Figure 3 shows the variations in the surface morphology of the SUS304 plate irradiated at different values of microwave power and irradiation time. The surface of the pristine sample transforms progressively into rolling hills with increase in the power and the time; however, the large protrusions and troughs are retained. The higher magnification images of these rolling hills reveal that columnar structures of several tens of nanometers in diameter covered the surface. Furthermore, nanostructures were not formed on the surface until 10 minutes of 500 W microwave irradiation. However, after 15 minutes of irradiation, a dense formation of the nanostructures was observed.

When microwave is irradiated to the metal substrate inside the reactor, an arc discharge occurs due to the short penetration depth of SUS304, and this leads to the generation of plasma and local surface heating. Oxidizing species such as O radical, O^−^, O^+^, etc. are generated from inside the plasma, and these oxidizing species causes the ion bombardment onto the substrate surface^48^. These harsh conditions lead to fast oxidation of the SUS304 sample surface, and the volume expansion caused from the oxidation become the driving force of the growth of nanostructures^49^.

It is noteworthy that the nanostructures fabricated using a higher power for the same duration of irradiation exhibited a larger average size and a lower number density. Owing to the antenna effect, the microwave power is concentrated on the local hillocks, and hot spots are created at the points, which make these spots to become molten and lumped. The nucleation starts at the molten hot spots^50^, and therefore, at higher power, the temperature of the hot spots increases, and the nuclei become larger. As a result, bigger nanostructures with a lower number density are formed at higher values of microwave power.

The hierarchical structures could also be prepared on uncleaned samples (without acid etching, see Supplementary Fig. S2), indicating that acid etching during the sample preparation is not the cause for the formation of hierarchical structures. The pristine SUS304 plate exhibited striped patterns on the surface originating from the continuous production process of the SUS 304 sheet. These striped patterns are the protruded regions that functioned as antennae to receive the microwave energy more efficiently. Therefore, these regions heated up more quickly and the hierarchical structures formed more quickly in these regions. However, as the microwave irradiation progressed, the hierarchical structures spread and eventually covered the whole surface of the SUS304 plate.

**Figure 4:**
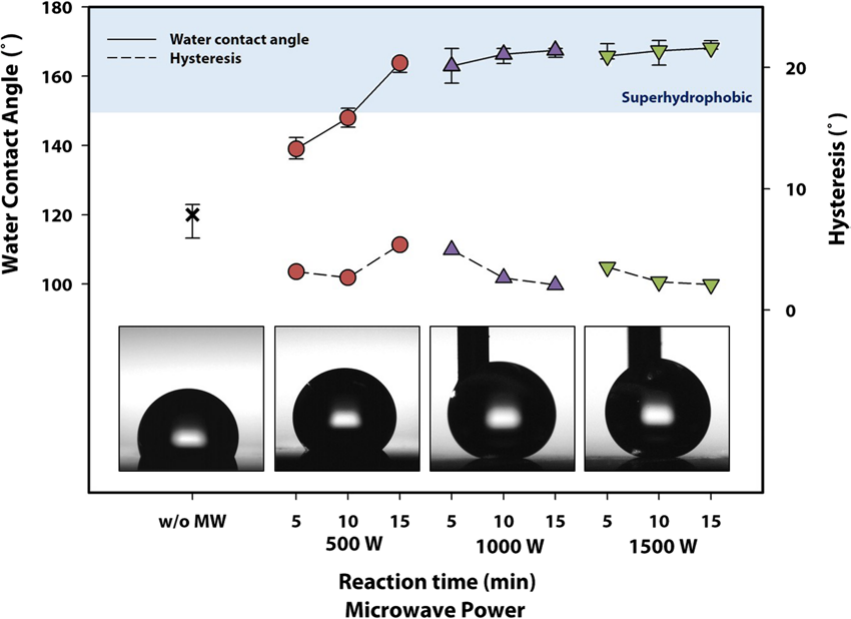
Variation in the water contact angle and hysteresis of the sample surface, prepared by irradiation at different values of microwave power and time, after the ODTS coating. The photographs in the insets show the shapes of the droplets on 10-minute irradiated surfaces at each microwave power, and the blue region shows the superhydrophobic region with CA > 150°. The water contact angle increased with increase in the microwave power and the irradiation time. The needles are shown in the images of the water droplets for 1000 W and 1500 W-irradiated samples because the water drops did not fall from the tip of the needles.

Figure 4 shows that a superhydrophobic surface can be obtained by simply coating the SUS304 plate with the hierarchical structure with hydrophobic ODTS. The sample irradiated using a microwave power of 500 W for durations up to 10 min exhibited low contact angles (<150°), since the nanostructures were hardly formed (which is evident from the SEM images in Fig. 3). On the other hand, very high contact angles (162~169°) with a very low hysteresis of up to 4° were observed for the surfaces with the hierarchical structures. It is to be noted that a superhydrophobic surface cannot be obtained by coating a flat surface (without the hierarchical structures) with ODTS or vice versa. For example, pristine SUS304 with a flat surface (except the large intrusions and troughs) exhibited a water contact angle of approximately 120° even after coating with hydrophobic ODTS. Likewise, microwave irradiation generates a hierarchical surface, but it is covered with oxide nanostructures that make the surface hydrophilic.

**Figure 5:**
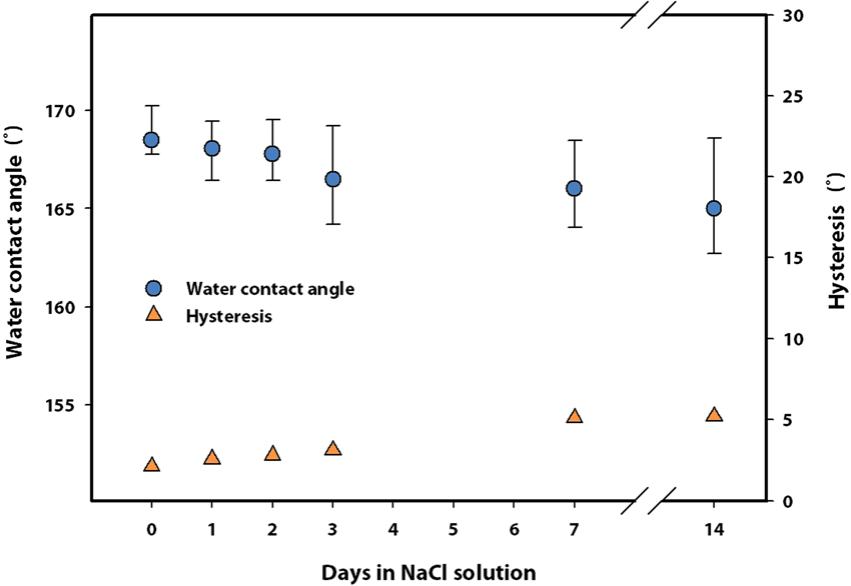
Water contact angle and hysteresis changing after soaking in 5% NaCl solution. The corrosion test was performed with 1500 W/5 min samples which showed the highest contact angle.

Corrosion test was performed in 5 wt% NaCl aqueous solution with 1500 W/15 min samples which showed the highest contact angle and lowest hysteresis among the samples. Figure 5 shows the water contact angle and hysteresis of the sample change by days in NaCl solution. The surface maintained superhydrophobicity after 14 days immersion to the solution, though contact angle was slightly decreased to 165° from 169° and hysteresis was slightly increased to 5° from 2°. That is to say, the surface can maintain superhydrophobicity in the electrolyte solution for more than 2 weeks.

## Conclusions

Hierarchical structures were fabricated on the surfaces of SUS304 plates by one-step direct microwave irradiation, and superhydrophobic surfaces (maximum contact angle of approximately 170°) were obtained after coating the surfaces with ODTS. The fabrication process is simple, fast, and clean, and the fabricated surface maintains superhydrophobicity even in electrolyte soution. The scaling-up of this method to a large surface is straightforward; a scanning microwave device can be used, which is readily available.

## Methods

### Sample preparation

SUS304 plates of dimensions 15 mm × 15 mm × 0.5 mm and 7.5 mm × 7.5 mm × 0.5 mm were cleaned by ultrasonication with ethanol followed by water. Subsequently, the samples were immersed in 50 ml of 1 M HCl solution for 3 h at 25 °C to remove the oxides on the surface. After the cleaning, the samples were washed with deionized water and fully dried in a vacuum oven at 120 °C for 1 h.

### Microwave irradiation

The details of the microwave irradiation system have been described previously^51^. The system consists of a magnetron, isolator, directional coupler, autotuner, quartz reactor, and gas feeders. The total pressure was maintained at 0.5 Torr during the microwave irradiation. Each sample was placed on the quartz plate at the center of the quartz reactor (30 cm long, 2.54 cm in diameter). After 10 minutes of purging with CO_2_ gas, the SUS304 plate was irradiated using microwave power (2.45 GHz, single mode) of 500 to 1500 W under the flow of CO_2_ (50 sccm) gas for 5 to 15 min.

### Hydrophobic coating

After the microwave irradiation, the sample was immersed in 10 ml of 0.1 mMol (octadecyl-tetrachlorosilane (ODTS) – toluene solution for 5 h and dried at 120 °C for 1 h to coat the surface with a hydrophobic self-assembly monolayer.

### Characterization

Scanning electron microscopy (SEM) measurements were carried out using XL30S FEG of Philips Electron Optics B.V. operated at 5 kV, and X-ray diffraction (XRD) measurements were performed using Rigaku D/MAX 2500. Transmission electron microscopy (TEM) and energy dispersive spectroscopy (EDS) analyses were carried out using JEOL JEM-2200FS with an energy-dispersive X-ray spectrometer operated at 200 kV. The water contact angle was measured with SmartDrop Lab using water drops of 5 µm diameter. To check the corrosion resistivity, the samples were immersed into 5 wt% NaCl aqueous solution for 1~14 days, and dried with vacuum oven to measure contact angle.

## Electronic supplementary material

Supplementary Video

Supporting information
