# Supplementary material for: Facile fabrication of superhydrophobic surfaces with hierarchical structures
Source: Sci Rep. 2018 Mar 6;8:4101. doi: 10.1038/s41598-018-22501-8 (PMC5840334; doi:10.1038/s41598-018-22501-8)
Supplement: Supplementary file 2 — Supporting information [file 41598_2018_22501_MOESM2_ESM.doc]

**Supplementary Information**

**Facile fabrication of superhydrophobic surfaces with hierarchical structures**

Eunyoung Lee^1^ and Kun-Hong Lee^1^,*

^1^Department of Chemical Engineering, Pohang University of Science and Technology, 77 Cheongam-Ro, Nam-Gu, Pohang, Gyeongbuk, South Korea

**^*^**ce20047@postech.ac.kr

**Supplementary Figure 1. TEM images of the nanostructures**

Nanostructures were peeled off from the surface by scratching the surface with a knife and dispersed into ethanol before TEM sampling. Images in figure s1 are from 1000 W / 10 min sample.


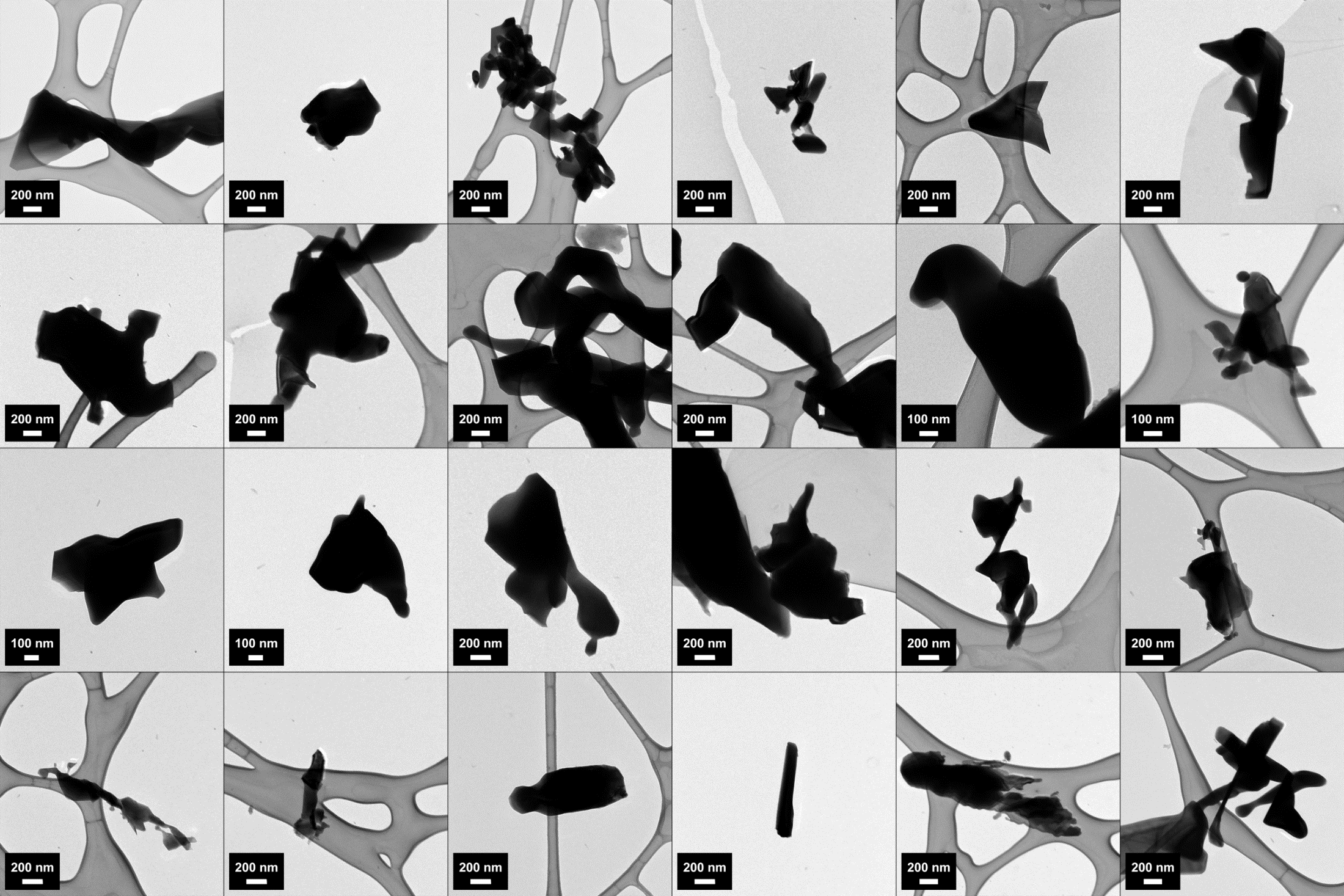


Figure S1. Images of various nanostructures from the surface of 1000 W / 10 min sample

**Supplementary Figure 2&3. Hierarchical structure from non-acid-washed samples**

Hierarchical structures were also fabricated from non-treated stainless steel 304 sheets. Micro-scale structures with nano structures were created as the surface got irradiated by microwave. When reaction time was increased, more micro structures were formed, while larger micro structures were formed with higher microwave power. Micro structures covered all over the surface after 15 minute reaction, and the surface structure became hierarchical. With higher microwave power, larger micro structures were formed. Roughness of the surface was increased with higher microwave power and longer reaction time. Roughness of non-treated surface was about Ra=150 nm, while 15 minute reacted sample with 1500 W microwave power has roughness about Ra=1078 nm.


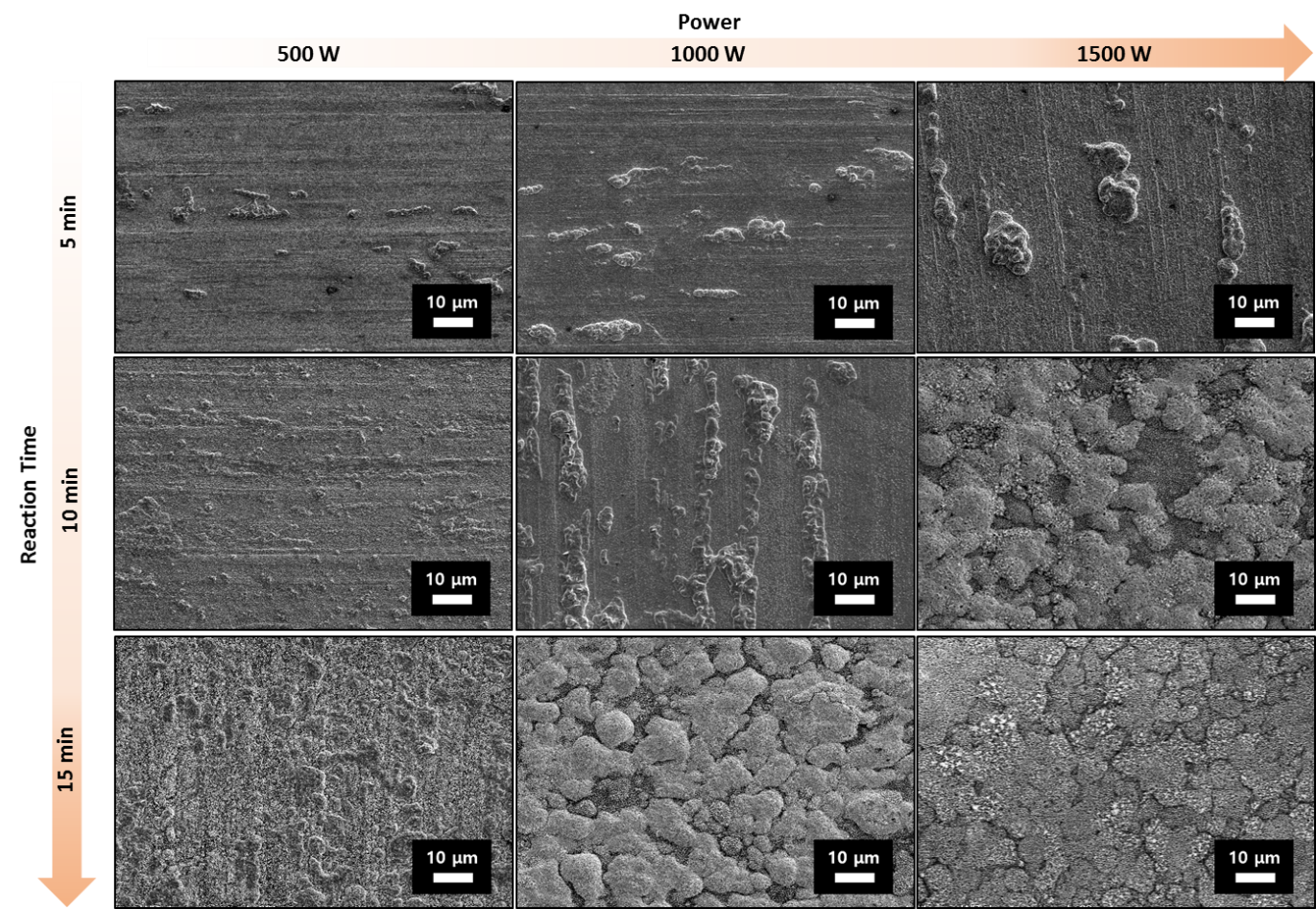


Figure S2. SEM images of hierarchical structures fabricated on the non-etched surface.


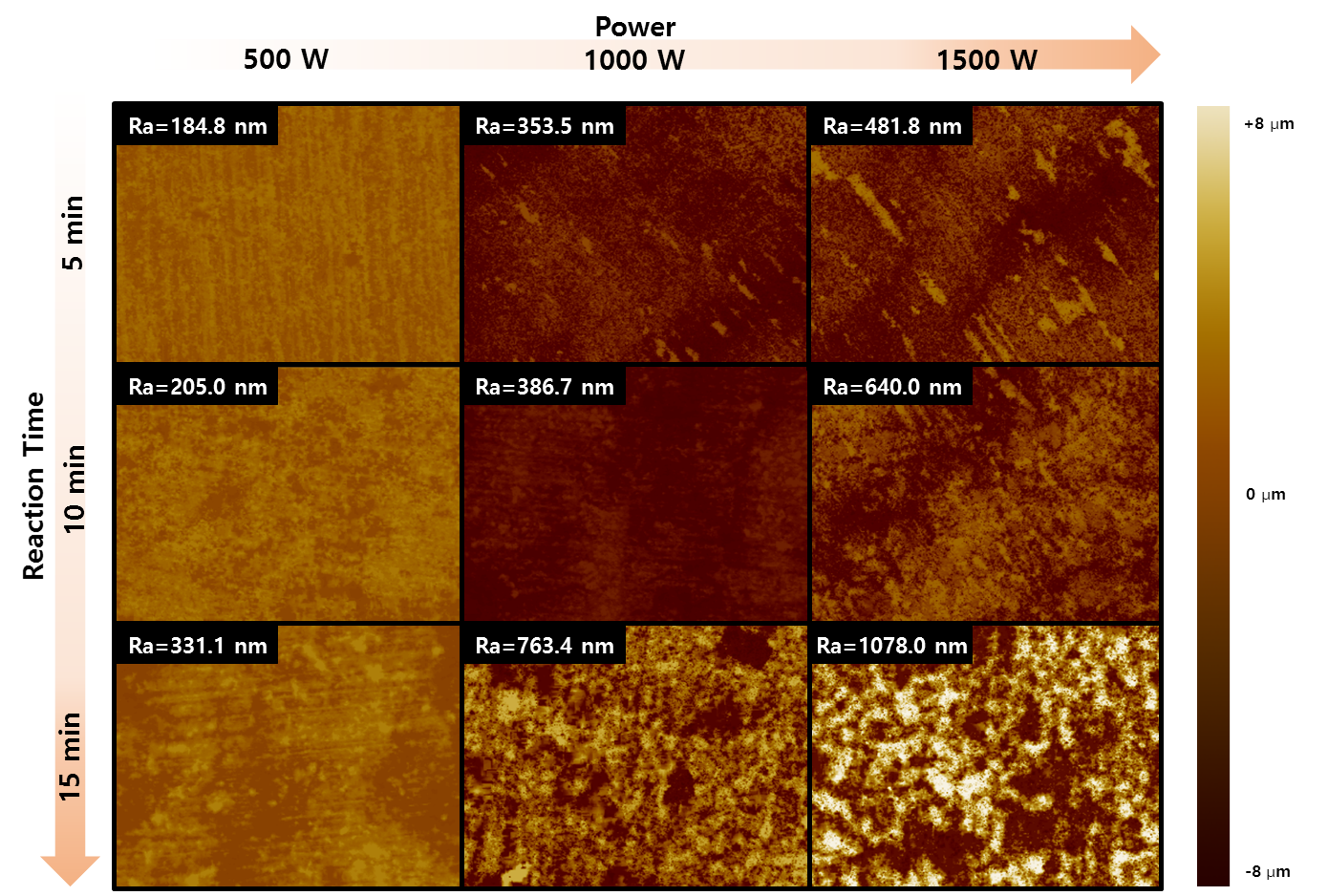


Figure S3. Surface profile images and roughness analysis of the non-etched surface. Roughness were increased by increasing microwave power or reaction time. Size of the images are 297 μm x 223 μm each.

**Supplementary Figure 4. Increasing contact angle with increasing surface roughness**

Increased roughness affected the water contact angle with the coated samples. After acid washing, only micro structures were formed, while only nanostructures were formed when the stainless steel sheet was irradiated with microwave under Ar+O_2_ atmosphere. With only nano- or micro- structures on the surface, contact angle about 120° was obtained after hydrophobic coating. However, hierarchical structures fabricated by microwave irradiation under CO2 atmosphere showed superhydrophobicity with water contact angle higher than 150°. Figure S4 shows the trend of water contact angle increasing with increasing roughness of the surface through different surface treatment.


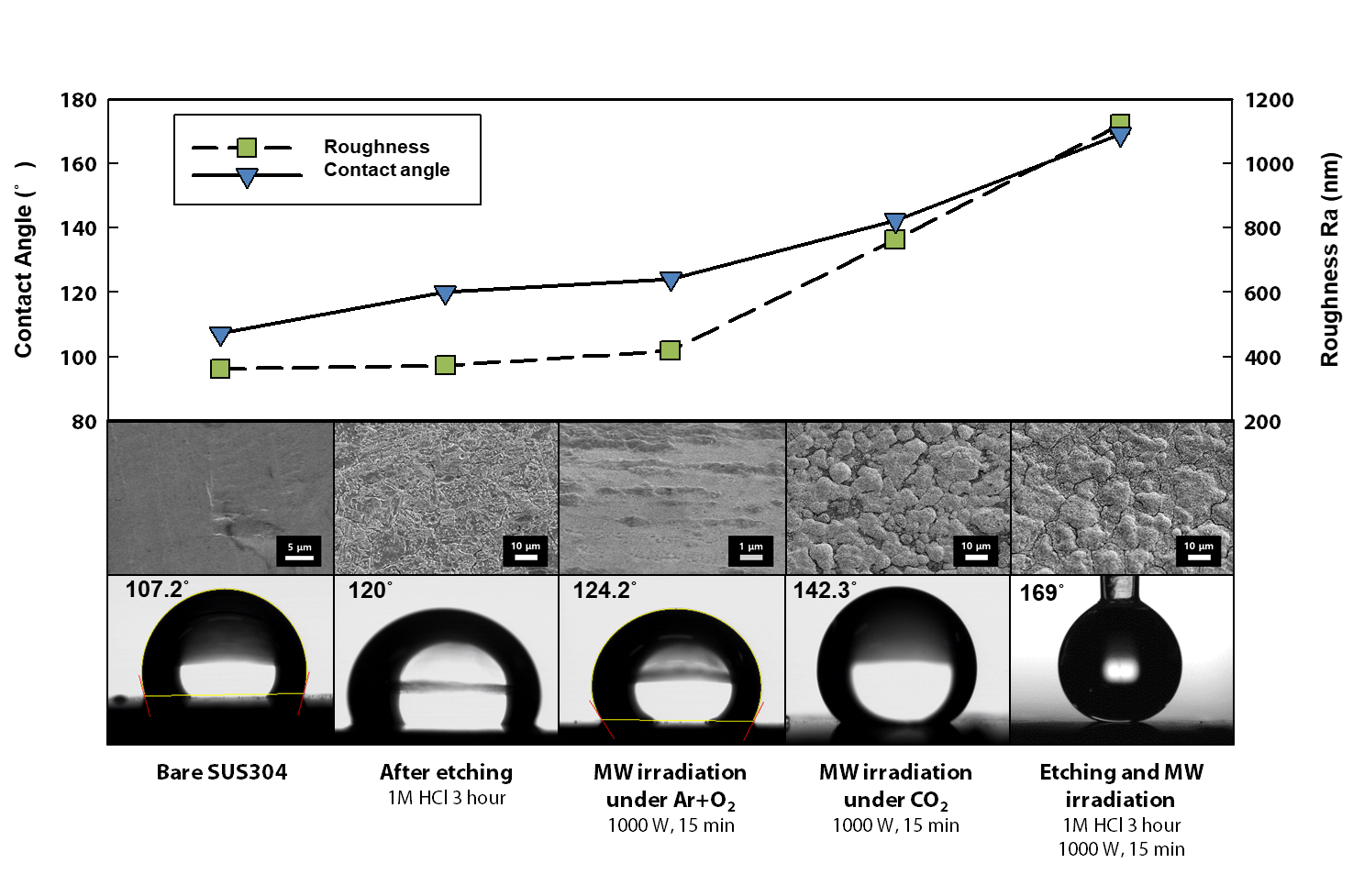


Figure S4. Trend of water contact angle and surface roughness with different treatment to stainless steel 304. Contact angles were detected after ODTS coating after each treatment has been done.

**Supplementary Figure 5**. Close-up images of the surface


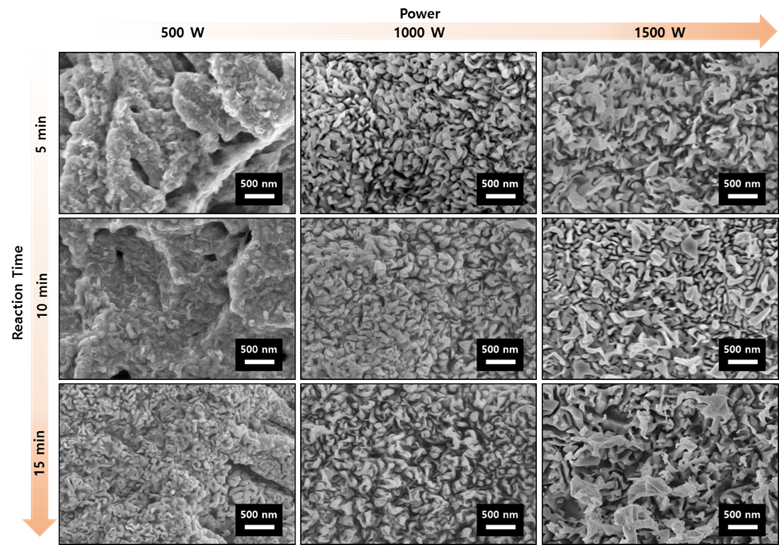


Figure S5. Close-up SEM images from insets in figure 3.
